# Bibliographical Notices

**Published:** 1838-07-01

**Authors:** 


### Periscope;

OR,

### CIRCUMSPECTIVE REVIEW.

" Ore trahit quodcunque potest, atque addit acervo."

### Notices of some New Works.

An Experimental Inquiry into the Physiology of Cutaneous Absorption, and its Application to Therapeutics, &c. &c. By *W. H. Madden*, M.D. 8vo. John Carfrae and Son, Edinburgh, 1838.

To this essay the gold medal was awarded by the University of Edinburgh, in August, 1837. Such a testimony forms ample passport to public attention;—and, indeed, the work displays very laudable research, and much soundness of judgment in every page. The Essay is divided into four parts:—the *first*, comprising the anatomy of the skin—the *second*, absorption from the uninjured skin—the *third*, endermic absorption—the *fourth*, the agents of absorption, and the therapeutical application.

For obvious reasons we will pass entirely over the first division—nor will we dwell very long on some portions of the second. This division opens with a chapter on "*Absorption in the Bath.*" Most of the arguments *against* cutaneous absorption have been drawn from this source, and therefore our author has "carefully reviewed all that has been already written upon the subject"—preferring the charge of prolixity to the hazard of leaving his task imperfect. The results of our author's own experiments are in favour of those who have advocated the power of the absorbents to suck up moisture from local application of warm water to the surface. Ancient and modern authorities are cited—from the baths of Nero's wife (Poppeia) down to the sailors in the launch with Captain Bligh—to prove general absorption of fluids by the skin. Our author made numerous experiments on himself as well as others—and all corroborated the opinion that fluids are absorbed by the skin.

"The balance which I employed was extremely sensible, vibrating perceptibly with a weight of a very few grains; and in order to obtain the greatest possible accuracy in my results, I made use of the following plan. Having carefully noted the spot to which the extremity of the beam, when perfectly horizontal, pointed, this was taken as the centre of a scale, constructed with the greatest care, by adding weights alternately to the beam and the seat, and marking the respective points which the beam indicated. By this means I was enabled to measure with the greatest accuracy any weight not less than gr. x., but lower than this I did not attempt to go, nor do I think that it was necessary. The scale then being affixed to the wall, and the balance so arranged, that the extremity of the beam pointed exactly to the horizontal line, I was accurately weighed, and the same process was repeated in half-an-hour. Immediately afterwards I entered the bath, and to avoid the most distant chance of fallacy from pulmonary absorption, my head was enveloped in an oiled cloth bag, to which a long glass tube was attached, and passed out of window, so that I breathed the external air alone. After remaining immersed for the same length of time, I was carefully dried, and again weighed. The results of twelve experiments thus performed, though not uniformly the same, were yet upon the whole extremely satisfactory." * 59

A chapter is dedicated to absorption of water from the atmosphere, and the result of observations and experiments are in favour of the said absorption. Dr. Combe is of opinion that the lymphatic temperament of the Dutch is owing to the absorption of moisture from the aqueous atmosphere.

"Upon the whole, then, we are, I think, justified in concluding, that the facts now related certainly constitute additional evidence in favour of the existence of this function of the skin. But at the same time, if we consider the numerous cases in which the body loses weight, even in a moist atmosphere, and the very few instances of augmentation which have been recorded, it must be admitted, that it is under particular circumstances only that the aqueous vapour contained in the circumambient air finds its way by this course into the system." 75

The third chapter on the absorption of miasmata, exhalations, contagion, &c., contains nothing important. The channel through which miasmata and morbid poisons are conveyed into the human system is not positively ascertained; but it is far more likely that they are taken in by the mouth than by the skin, notwithstanding some ingenious reasons for the latter supposition adduced by our author. As for the poison of syphilis, no man in his senses can now doubt that it is frequently, perhaps most frequently, absorbed from an unabraded surface. The absorption of morbid poison from glandered horses is attested by Elliotson and others.

The fifth chapter is on the absorption of medicinal agents. We have devoted several articles in this Journal to the endermic treatment of diseases that we need not dwell long on this chapter. The application of mercury to the surface, and its absorption into the system is one of the most familiar and convincing experiments—though some physiologists, even to this day, maintain that no absorption takes place, but that the nerves are the agents in this affair. We need not allude to the absorption of iodine, antimony, arsenic, lead, &c., as the facts are incontestible. We are much in the habit of using tartar-emetic externally; and have often observed that sickness of stomach resulted. Most people are in the habit of rubbing croton oil on the abdomen in cases of obstinate costiveness, and with good effects. Dr. A. T. Thomson has found jalap and lard rubbed on the stomach produce griping and purging—and that poultices of rhubarb will act on the bowels of children.

"*Experiment.*—July 19, 1836. At 12 P.M. I applied to my abdomen a large poultice of oatmeal, mixed with about ʒvi. of jalap in powder. It excited such intolerable itching, that I could not sleep, and at 5 A.M. I removed it. Notwithstanding this, however, my bowels were very freely opened next morning."

Opium, belladonna, and other anodynes prove not so clearly absorption, as some other substances, since the effects may be attributable to their agency on the nerves.

The second section of this division is on the absorption of liquids; but it is scarcely necessary to accumulate evidence of the absorption of liquids, when it has been abundantly proved as to solids. Speaking of iodine, the author thinks friction with the tincture is far better than when in combination with lard or any oily substance. The iodine or biniodide of mercury combined with soap liniment will, we apprehend, prove a useful external application, as well as internal. Dr. Madden made experiments on himself with iodine, and detected the medicine clearly in the urine. We must conclude this short notice of Dr.

* "The whole of these bath experiments were performed in August and September 1836, the weather being for the most part fine, and the air of moderate temperature."

Madden's very laborious and useful researches, with the following extract or resumé.

"It only remains, therefore, before bringing my labours to a close, that I should add a few words by way of resumé. In the foregoing pages, we have traced the remarkable analogy presented by the tegumentary organs throughout the animal kingdom, in all its gradations, from the highest to the lowest. We have seen that, while varying greatly in degree and extent of development, they have yet, in accordance with the beautiful and uniform simplicity of nature, been all, or almost all, constructed upon the same general plan; and we have hence argued, that since cutaneous absorption is undoubted in the lower tribes, we might fairly assume that it is not altogether wanting in the higher. Proceeding still farther, we have shewn, in opposition to the assertions of many physiologists, that the human epidermis, in place of being a mere inorganic varnish, spread over the surface of the true skin, to protect it from injury, and obstruct the passage of deleterious agents, is a membrane of a somewhat complex structure. We have seen that even if it does not possess a set of vessels peculiar to itself, it has yet embedded in its substance loops of the subjacent plexus of lymphatics, this arrangement being doubtless adopted for some specific purpose. We have shewn that it is not impermeable to fluids, and we have advanced reasons for distrusting the opinions of those who hold it to be destitute of vitality.

Fortified by these anatomical preliminaries, we have gone forward in our inquiry. We have found that various parts of the body, when steeped in fluids of a bland unirritating nature, attract to themselves an appreciable quantity; and that the rate of this imbibition may, to a certain extent, be determined by actual admeasurement. We have observed the *absolute* increase of weight which follows immersion in the warm bath, when no possibility of pulmonary absorption could exist, and at the same time we have endeavoured to prove, that the danger of fallacy from this source has been greatly exaggerated. We have seen thirst removed by the same means, and have reviewed those singular instances of long-continued abstinence from drink, of excessive and prolonged discharges, and of suddenly augmented weight, which can be explained upon one supposition only, viz., absorption from the atmosphere. We have next drawn arguments from the introduction of malaria into the system; we have proved the absorption of putrid exhalations, and shown how strongly the doctrine of contagion supports our proposition.

Nor does our evidence end here. By numerous and diversified observations, and by unequivocal experiments, we have demonstrated the important fact, that medicinal agents, when applied to the uninjured skin, be their form what it may, be they solid, liquid, gaseous, or in vapour, will speedily reach the interior of the body, and there exercise their accustomed actions. In this manner we have seen pain alleviated, and disease removed. We have seen death produced by the external application of a poison. Nay more, we have followed a substance thus placed in its progress inwards; we have detected its peculiar odour in the secretions; and, finally, we have shewn its presence, by the almost unerring test of chemical analysis.

Can more than this be required? Surely even the most sceptical, after an impartial consideration of these proofs, can scarcely persist in refusing his assent to the existence of cutaneous absorption. Unless utterly immoveable by testimony, he must at length be compelled to acknowledge that, if all this be true, the evidence is overwhelming; that the doctrine rests upon as sure a basis as we can ever hope to obtain in physiological inquiries." 151.

We recommend this little work to the library of every medical man who wishes to know all that has been said and done on the subject of cutaneous absorption.

---

NOTES ON THE MEDICAL TOPOGRAPHY OF CALCUTTA. By *James Ranald Martin*, Presidency Surgeon, and Surgeon to the Native Hospital. Printed by order of Government. Calcutta, 1837.

It is now about 150 years since Job Charnock pitched his tent at Calcutta—chiefly because there was a large shady tree there, under which he might smoke his hooka in cool contemplation. It now contains nearly 70,000 houses! Mr. Charnock, however, chose a very bad locality for the emporium of commerce and future Metropolis of the East. In fact, he could scarcely have selected a more malarious spot between the Himalayha mountains and the sea-board of the Sunderbunds!

Upon the moral, physical, and political condition of the inhabitants of Calcutta, European, native, and foreign, Dr. Martin makes numerous and sensible remarks, which must be very interesting to our oriental countrymen, but which we cannot dwell upon here. The work must have cost the author immense pains, as it goes most minutely into all the details and ramifications of medical topography—and not only so, but into the nature and treatment of the endemic diseases of the place. We here present an extract, which we think Dr. Dickson of Cheltenham (the professed enemy of the lancet in all tropical diseases) ought to peruse with serious meditation on the tendency of his doctrines, should they ever have influence on the mind of the tropical practitioner.

"It only remains to notice the prevailing treatment of the dysentery of Bengal, amongst the more experienced practitioners at the Presidency, and this I shall insert in the order of importance. Blood-letting, general and local, as first practically urged in the dysentery of India by Dr. James Johnson, takes the lead, and has done so for many years: it is the standard remedy; and I believe that when the subject comes early and freely under this treatment, and that the case is not complicated with hepatic congestion or actual disease, little else than a few purges and sudorifics will be required for the cure; but as in most cases of this formidable disease, as it appears within the tropics, the diseased state of the large intestines is essentially mixed up with general abdominal engorgement, other and important means immediately follow the bleeding; and of the first are those which act powerfully on all the secreting organs, internal and external. Calomel in full doses with antimony, or with ipecacuanha, followed by purgatives, sudorifics, warm baths, enemas and other minor adjuvantia. I believe this to be the general course here." 116.

The following passage appears to be prophetic, and could not have been more to the point, if Dr. Martin had been sitting at Dr. Dickson's elbow while the latter *Gentleman* was inditing his philippics against Dr. Johnson's work on Tropical Climates.

"The above quotation I have made from the work of Dr. James Johnson—a gentleman to whom the Indian military surgeon is under a weight of obligation, which I regret to think that *any one* should be found unwilling to acknowledge, or disposed to return with any other than grateful feeling." 95.

The following passage possesses interest for the European as well as the tropical practitioner—seeing the great number of tropical invalids which annually arrive on our shores from the burning skies of the East.

### CHRONIC ENLARGEMENT OF THE LIVER.

"This disease is by no means uncommon in Bengal, as a sequel to fevers both remittent and intermittent. The function of the organ is greatly impaired; there is frequently a hacking dry cough—dyspepsia in various forms, and general ill-health, with a sallow pasty complexion and emaciation. The treatment of this disease is not well understood. Mercury I believe to be injurious: it injures the stomach and bowels—already over drugged, without exciting any secretion from the organ chiefly affected, and on which mercury, from repeated use, has lost its effect:—purgatives of an irritating or drastic nature are equally injurious; in fact, it is often an unmanageable disease, not readily amenable to treatment or change of climate.

The plan of treatment I have generally had recourse to in such cases is the nitro-muriatic acid bath, steadily persisted in for a month or six weeks at a time: it seems, like mercury, to act powerfully on all the secretions, and in the cases here spoken of, I do not know a better remedy. When from morbid dryness of the skin, the absorbents will not readily take up the acid, I direct the occasional use of the vapour, or warm water bath, with powerful friction of the whole surface, in order to stimulate and purify the skin." 135.

Directions for Using the Bath.

"1st. Two gallons of water (about ten bottlesful) may suffice for a bath.

2nd. To each gallon of water add 3 oz. of the dilute nitro-muriatic acid by measure.

3rd. The bath thus prepared will keep in use for three days, by adding half an ounce of the dilute acid and a pint of water, morning and evening, in order to make up for the waste by evaporation.

4th. A portion only of the bath to be heated for use, after which it is to be added to the remainder, so as to make the whole of a comfortable warmth.

5th. Let both feet be placed in the bath, while the inside of the legs and thighs, the right side (over the liver) and the inside of both arms are sponged alternately: this should be continued ten or fifteen minutes morning and evening.

6th. While using the bath, a gentle aperient, such as Cheltenham salts, or Epsom salts in some bitter infusion, should be taken every other morning.

7th. Earthen or wooden vessels should be preferred as foot-baths, and all the sponges and towels to be kept in cold water, as the acid corrodes them." 138.

The code of hygienic maxims for the guidance of European sojourners concludes the work, which is constructed with great labour, talent, and judgment. It presents an admirable specimen of what a treatise on medical topography ought to be. Dr. M. deserves the thanks of all ranks in Bengal, from the Governor General to the palankeen bearer.

---

The Reform of Criminals and of Prisons. 8vo. pp. 20. Stephens, Bell Yard, March 1838.

The title of this little pamphlet may induce many of our readers to suppose that the work can have little to do with the practice of medicine. But this supposition would be erroneous. The study and the practice of physic include almost every thing that affects the morals and the happiness, as well as the health of the community.

"Quicquid agunt homines—votum, timor, ira, voluptas."

All come within the pale of the medical philosopher's contemplation and influence. The medical man mixes with all classes of society; and being considered as a person of education, and well acquainted with human nature, his opinions are listened to with respect, and treasured up with care. Besides, there are medical officers to all Prisons, Jails, and Penitentiaries—and there they have good opportunities for observation, and ample range for the exercise of their knowledge.

The pamphlet before us is a very sensible one—and is evidently the production of a man well versed in the operation and tendencies of our criminal codes —as well as of our receptacles for the punishment or confinement of law-breakers. The abolition of the punishment of death, in all except atrocious crimes, is a modern triumph of justice and humanity over the most sanguinary and inefficient code that ever disgraced a legislation or stained a civilized people! Severe to the criminal, it was indulgent to the crime—and offences were *encouraged*, because the rigour of the law led to impunity in most cases, and uncertainty in all!

Corrective punishment has hitherto failed in this country—*first*, because it was not calculated to reform the character of offenders—and *secondly*, because discharged prisoners had no chance of honest employment—no resource for subsistence but renewed crime! It is the brand on his character, not the bodily infliction, that the prisoner has to dread. The period of incarceration is not the limit of punishment, as many thoughtless magistrates seem to imagine. It acts with few exceptions, as an endless proscription from every respectable pursuit in this country. Until prisons shall be constructed and managed so as to purify and reform offenders, the sentence, in its main operation and effect, may be considered as *for life!*

"Were the increase and diffusion of moral evil the aim and intent of punishment, if the sentence prescribed 'imprisonment, with instruction in criminality by experienced artists,' no other preserves of vice than these prisons could be desired for the purpose of its dissemination over the untainted parts of the community." 7.

This is a frightful, but we have no doubt, a faithful picture of the effects of "Prison discipline!" A vicious gaol infects not merely its immediate neighbourhood, but diffuses the moral leprosy over a wide circle of society.

"Every gaol should provide as means essential to the reform of offenders and the protection of the public,

1st. The separate confinement of each prisoner.

2nd. His religious and moral instruction.

3rd. His instruction in some useful mechanical art or calling.

And Government should provide,

4th. Encouragement to the emigration of prisoners on their enlargement.", 10.

It has been customary, of late, to rail against the inhumanity of solitary confinement; but by "separate confinement" our author does not mean seclusion without employment.

"As a separate system is designed to act chiefly by its corrective influence on the mind, it requires for its salutary administration, a vigilant superintendence by more discerning visitors than turnkeys. Well executed, it will be found an effectual, and in most cases, the only effectual engine of reform. When the prolonged endurance of it leads to unfavourable results, the prisoner should be placed in a class upon the silent system, which is the best substitute for the separate." 11.

This system preserves the prisoner from the contamination of associates more advanced in crime, and from the formation of friendships and acquaintances that might be injurious to him on his return to society. It is to be conducted under the resources and supports of religious and mechanical instruction, with proper attention to health by ventilation, temperature, exercise, and proper diet. The pamphlet is well worthy of perusal by every man interested in the happiness of his species.

---

MEDICAL PORTRAIT GALLERY, &c. By *T. J. Pettigrew*, Esq. to be continued monthly. Price 3s. Fisher, &c. Three Portraits, 1838. Parts 1, 2, 3.

Cotemporary, or living biography has never, we believe, succeeded to any great extent. The reason is obvious. The lights and shades of living characters cannot be faithfully portrayed, without great risk and inconvenience—except anonymously—and then it is generally caricature. When the biographer is acknowledged, the character of the individual is, almost necessarily, overlaid with eulogy; the virtues are all placed in the foreground of the picture—and the defects in the shade. It is very different when "the dull cold ear of death" no longer listens to panegyric or censure. The biographer may then delineate the character, praise the virtues, censure the vices, and criticise the writings of the departed, without fear or reproach. There is also another disadvantage in contemporary biography :—the life is not finished—the last act of the drama—often an important, and always an interesting one—has not been enacted.

Still, with all these drawbacks, living biography is not without its utility, and even its attractions. We greedily peruse the various incidents in the lives of those who have figured on the stage, when the curtain drops, and it is not unnatural that we should have much curiosity to learn the chief particulars, however varnished by the pencil of flattery, while those characters are moving around us, and playing their parts in the many-coloured scenes of human existence. The living biographer must necessarily draw the chief incidents and epochs from the individual himself, whose life is placed on record, and thus the work becomes a kind of auto-biography—a species of history which has, in all ages, been greedily devoured. The work now commenced by Mr. Pettigrew, has also this advantage, that two thirds of it will consist of *post-mortem* biography, and consequently be free from the objections inseparable from the portraiture of living and contemporaneous characters. In the *former* and major part of the undertaking, it only requires industry, research, and discrimination :—in the *latter*, it requires great skill, intrepidity, rigid impartiality—and last not least, a stern abhorrence of pandering to the cupidity, vanity, or fears of those whose portraits are placed on canvas. Most of us remember the scandalous use, or rather abuse, that was made of this branch of literary labour by the late Dr. Nesbitt, whose poverty was but a poor excuse for the mercenary trade which he carried on. He went round to the various eminent personages in our profession, soliciting particulars of their lives, and hinting pretty unequivocally that they were to be idolized or victimized, according to the amount of the *subscription* for carrying on this nefarious *work*. We learnt, at the time, some curious anecdotes of characters, some living and some now dead, who were betrayed into pretty considerable sacrifices of purse, from fear of this speculative trader in fulsome praise and vindictive calumny. We have no apprehension indeed, that the biographer before us will condescend to any of the mean and mercenary arts of his predecessor; but we shall be acting the part of friends, in warning him against rendering himself liable to the slightest suspicion of such derogatory procedure, by unmeasured, or unmerited censure or *flattery* of the living portraits. The latter rock is the more dangerous of the two. Upon it are syrens sitting to lure him to the fatal shore—warblers—

"Whose song is death, and makes destruction please."

The first part of the Medical Portrait Gallery has produced a favourable impression on our minds, not unalloyed by some fears, for the execution of the dangerous and difficult department of the work.

The first number contains three portraits and biographical memoirs. The first—"Esculapius"—gives the Editor an opportunity of shewing his Egyptian and oriental lore to advantage; but, as the character is fabulous, we shall pass it over. The memoir of Albinus occupies only two pages of letter-press. This distinguished anatomist was born at Frankfort in 1697—taught anatomy nearly fifty years in Leyden, and died in 1770, aged 73 years. An elegant portrait, and an authentic autograph of this great man embellish the short memoir.

Next to the elaborate dissertation on Esculapius and the fabulous æra of medicine, we have the biography of Sir Henry Halford, occupying fourteen quarto pages, with a most striking likeness, beautifully executed. Considering the position in which the subject of the memoir stands, and the extensive influence which he wields amongst the very highest classes of society, whether in the senate, church, at the bar, or in the profession itself, we must acknowledge that the living biography of such a man is as free from fulsome adulation as could possibly be expected under existing circumstances. It is true, that in the portrait there are no specks—no traits of human frailties: but still the language of panegyric is subdued, and the greater part of the memoir consists in a review—a favourable one, of course—of Sir Henry's scattered essays and orations.

This distinguished physician—the son of a physician, Dr. Vaughan—was born in Leicester, Oct. 1766—and consequently is in his 72d year. He is a remarkable instance of the degree of energy, both mental and corporeal, which may be retained beyond the tenth septenniad! Sir Henry is just as active a practitioner at this moment, as he was twenty years ago, when we first became personally acquainted with him. Educated at Rugby and Oxford, Sir H. became an excellent classical scholar, and so retentive is he of all the grammatical niceties of the Greek and Latin languages, that, the last time we met him in consultation, we found him amusing his vacant *moments*—that is between the houses of his patients—by translating English ballads into Sapphic verse—verse which Horace would not have disowned in the Augustan age! Having contracted a matrimonial alliance with a lady of noble race, he soon became the fashionable physician of the day—probably more owing to the elegance of his manners, the extent of his learning, and the influence of his connexions, than to any superiority over his contemporaries in practical knowledge or pathological research. Indeed he got into high practice before the age of thirty—and never having been attached to any hospital, he could not, at that age, and especially at that æra, have had much clinical experience, while pathology was almost unknown. Sir Henry presents one of the very few instances in this metropolis, of a physician penetrating into the very highest circle of practice, and consequently reaping the largest harvest of wealth, without being a medical officer of any hospital. Babington, Baillie, Curry, Warren, Pearson, Nevinson, Chambers, Elliotson, &c. &c. might be cited as illustrations. But Sir Henry outstripped all his compeers, with all the advantages of their clinical knowledge and patronage of pupils. We do not wonder at it. A notion prevails among those who are not acquainted with Sir Henry that, from his long course of attendance in the chambers of the high nobility, where imaginary are more numerous than real diseases, his practice was milk and water, and rarely active or decisive. This is an error. The worthy Baronet is by no means deficient of energy, where the case requires it—and his long experience enables him to detect the nature of a malady very readily, and grapple with it stoutly. It is very true that, for obvious reasons, this eminent physician has not been able to keep pace with the progress of pathology; and consequently he is defective in his diagnosis of diseases of the chest, whether cardiac or pulmonic. This deficiency, though it might be an awkward inconvenience in the wards or dead-house of an hospital, was of very trifling import in the rounds of private practice, where it was seldom suspected by the friends of the dead, and still more seldom demonstrated by the scalpel of the anatomist. But had this retardation in the march of pathology been ten times more patent than it was, it would have been amply compensated by the *tact* of this gifted physician, unequalled in any age or nation! We sincerely believe that, since the days of Esculapius himself, down to the present moment, no medical practitioner ever entered the room of sickness so well calculated to cheer the drooping spirits, raise the hopes, or inspire the confidence of the patient, as the physician whose biography is here undertaken! The smiling countenance, the courtly manners, the eloquent language, wearing the appearance, if not the reality, of the most sympathising friendship, the firm tone, the artful but judicious interrogations, the patient, attention to garrulous details, the perfect mastery over every symptom of doubt, much less alarm—all combined with numerous other traits of bearing and conduct which language cannot convey, to impress the sufferer with a conviction that, if the disease admitted of remedy, here was the man most qualified by nature and art to apply it. We have, on more than one occasion, heard patients remark, after Sir Henry had left the house, that they would almost as soon die under such a physician as recover under some others whom they could name! This, though the chief, is not the only qualification which Sir Henry possesses as a powerful passport to fame and favour in the eyes of the world. His resources are absolutely inexhaustible. Not only can he vary the daily physic, with surprising ease, but he can gratify the most languishing and fastidious appetite of the invalid with innumerable forms of diet, which would seem to have required half one's life to study and manipulate. By the magic wand of this accomplished physician, the most protracted, painful, and incurable maladies are converted into a kind of luxurious anticipation of daily relief from medicine, and hourly enjoyment of the palate. We are, at this time, attending an octogenarian, whose taper of life has been kept, for some months, from extinction by a glass of rum and milk prescribed by Sir Henry early in the morning, after which, the bed-ridden invalid falls into a delicious sleep of some hours, dreaming of early scenes, and awaking refreshed, with constant blessings on the head of the physician who ordered so delightful a cordial for the decaying frame! But Sir Henry's resources and endowments are not confined to physic or physics—they extend to metaphysics. The dying Christian would find in him a more cheering philosophy and religion than most of our divines would be able to convey to the spirit retiring from its earthly tabernacle to soar into other and unknown regions. Discourses have been delivered by the subject of our memoir which would have been more appropriate in the mouths of prelates, than some of their harangues in the House of the Lord—leaving aside their orations in a house of many Lords.

With such natural talents, acquirements, and *tact*, is it wonderful that Sir Henry should have been the favourite physician of three successive sovereigns, with their courts, camps, senates, and aristocracy? The urbanity of his manners, the polish of his conversation, and the honorable etiquette of his conduct amongst his brethren, have rendered him a favourite consultant in all cases, and secured his professional popularity up to the eleventh septenniad of his life. We have met him when we were at "daggers drawn" with the College over which he has so long presided, and when our professional existence was in some danger from the anathemas of that once powerful, but now very harmless, body of medical aristocrats; yet never experienced the least deviation from friendly intercourse and professional equality. From Sir Henry Halford we never received or solicited either favour or patronage—and never will. We have drawn up the foregoing sketch of his professional character, from twenty years' observation of it, and without referring at all to the memoir of Mr. Pettigrew. We have neither extenuated the defects nor exaggerated the virtues of this distinguished physician, being perfectly independent of his frowns or favours; but only desirous of exhibiting the prominent traits of character which we have observed, without the slightest bias from medical politics or personal considerations.

The Second Part contains Portraits and Memoirs of Ruysch, Haller, and Sir A. Carlisle—all ably executed—but the last one not so good a likeness as we expected; but it was painted many years ago, and Time produces great change of features!

With the works of Ruysch the profession is well acquainted. He was born at the Hague in March 1638. Studied at Leyden, where he graduated—and settled in his native town. The plague having broken out in Holland, Ruysch was appointed by the States General to attend the sick at the Hague. He afterwards devoted himself to anatomy, human and comparative, and, as is well known, made great progress—and perhaps discoveries. His vascular injections have rarely been equalled or surpassed. His time was so much taken up in the actual prosecution of anatomy, that he had little time for reading, and therefore often made real discoveries which had been made before, but were unknown to him. This, of course, led him into controversies, such as we have now, in full vigour, regarding the "excito-motory" function and nerves. He was Professor of Physic for forty-three years, when he fell and fractured his thigh-bone, which disabled him for further exertion. He died of fever in 1731, aged ninety-three years.

Of Haller we need not speak, his works and his life being equally well known to the world. The memoir of Sir A. Carlisle is extended to fourteen quarto pages, and the particulars of his life, as well as the multifarious productions of his pen, are pretty minutely stated. Sir Anthony is now in his seventieth year, but he is not near so active as his contemporary and senior, Sir Henry Halford.

As one of Sir Anthony's productions will shortly come under notice in this Journal, we shall not, at present, advert to the somewhat eccentric character of this talented individual.

The Third Part contains portraits and biographical sketches of Lineacre, Akenside, and Sir C. M. Clarke, Bart.

Akenside is more known as a poet than a physician—and the two most remarkable traits in his character are, his shame of being the son of a butcher (a striking weakness of mind)—and his barbarous treatment of his patients in St. Thomas's Hospital. If he had had half as much magnanimity as he had imagination, he would not have eschewed the humility of his origin—and, at all events, he would have curbed his butcherly propensities to the afflicted poor under his rude rule in a public institution. We have no patience with upstart tyrants—ashamed of what they ought to be proud, (humble birth)—and proud of what they ought to be ashamed—power of abusing their stations!

But the Lion of the Third Number is Sir Charles Mansfield Clarke, with a retrospective glance at his elder brother, Dr. J. Clarke, whose flashes of merriment were wont to set the class-room in a roar, in Old Burlington-street, some thirty-five or forty years ago. We well remember the droll and dapper little body, every gesture of whose countenance was calculated to inspire good humour, and excite the student's risible faculties. But he passed from the scene, at the early age of fifty-six years, bequeathing to his brother, his wit, humour, talents—and, a *wheen* of patients into the bargain!

Sir Charles was born in 1782, and consequently is in his fifty-sixth year. He was a fellow-student of our own, in Great Windmill Street, under the late Mr. Wilson, and the present Mr. Thomas. Having served a short time in the army, he entered on practice under the auspices of his brother; and commenced lectures with him in 1804. Although a very different character from Sir Henry Halford, whom we have just sketched, yet a great deal of what we have said respecting the senior baronet will apply to the junior. But their professional avocations ran in very different channels. In an overwhelming majority of instances—in forty-nine cases out of fifty, the *lying-in* chamber is anything but a *dying-in* apartment. In the former, the doctrines of Malthus and Martineau are thrown to the winds, and the addition of every unit, doublet, or even triplet to the sum total of the population, is hailed with rapture, and announced with glee, throughout the puerperal mansion. Never was man or midwife better calculated to enjoy the hilarity of the scene, or augment its intensity, than Sir Charles Mansfield Clarke! This amiable and talented physician seems as though he had been born for beguiling the tedious hours of labour—*taking* the pains from the fair sufferer—and by his looks and language inspiring hope, and banishing every shadow of despondency. To an inexhaustible flow of animal spirits, Sir Charles adds the auxiliaries of wit, humour, anecdote, and the most joyous of countenances. But one remarkable and highly useful talent he possesses in an extraordinary degree—the happy facility of explaining and elucidating those numerous subjects which naturally occupy attention, and start inquiries in a parturient bed-chamber. Woman is always a curious and inquisitive creature; but when the *chef-d'œuvre* of Nature's works—the evolution of man—is in progress, her curiosity, and that of her attendants, are excited to the utmost stretch. On these occasions, the ingenuity of Sir Charles is never at fault. A screw, a wedge, a nut-cracker, a smoke-jack, or any of the most common implements, or familiar agents in mechanics, art, or science, furnishes his fertile brain with prompt illustrations of the complex and wonderful machinery which is in operation for bringing into this world an ample supply of human beings to compensate the havoc produced by the scythe of Time, the sword of war, and the ravages of disease. These, however, are only extrinsic, though important accomplishments in the lying-in chamber. Sir Charles has resources at command of a much higher order, and a more intellectual character. He had the good fortune of having an elder brother of prime talents, and large experience, who ushered him into practice, assisted him by his counsel, and shielded him with his name. If such inestimable advantages are comparatively thrown away on some people, it was not so with the subject of this memoir. Sir Charles was born with brains in his head, which is more than every one can boast of—and he did not put his brains into his pocket, whatever else may have found its way there. By means of a quick perception, keen eye, and inexhaustible energy, knowledge rapidly accumulated, and its valuable products were as rapidly diffused over an extensive field of practice. The consequences were, a princely fortune and an unbounded reputation, before the age of fifty years! Envy, too, which follows wealth and fame, almost as regularly as the shadow follows the substance, has not pursued the personage in question,—or, if it did, it skulked in holes and corners unseen, unheard. Sir Charles has been liberal to his brethren and they have been just towards him. Few other men could roll away to the coast or country, for five or six months annually—and return with a flowing sheet to a spring-tide of practice. Nothing but the unlimited confidence of the public, and the well-earned respect of the profession, would enable a medical man thus to sally alternately from the vortex of the metropolis to the "otium cum dignitate" of rural life, with undiminished influence or celebrity.

---

### The Philosophy of Marriage, &c. &c. By *Michael Ryan*, M.D. Churchill, 1838.

This, in the language of Yates of the Adelphi, is "a decided hit." In the whole range of bibliography, there is not a more "taking title," than the above. Old maids and decayed bachelors will peruse the work from curiosity. People who have been long in the happy state of wedlock will eagerly read it—wondering that they should have been *philosophers* so long, without knowing it—but to the boarding-school misses the "Philosophy of Marriage" will be an invaluable treasure. We all know what excellent smugglers these folks and their "*aides*" are, and we venture to prognosticate that the "Philosophy of Marriage" will be a "Stock-book" in every seminary of education between Kilda's Rocks and the Cliffs of Dover. How far it was prudent to address a work with such a popular title to the *general* as well as the professional public, may admit of some doubt. Hymen is represented with a torch in one hand and a veil in the other—clearly indicating that but a very faint light was to be thrown on the hymeneal mysteries, which were to be kept *veiled* from eyes profane. In the "Philosophy of Marriage," the veil is partly, if not wholly thrown aside, and the meridian sun is permitted to shed his profane rays on the inmost recesses of the conjugal chamber! The *generation* of animals and vegetables is displayed with physiological minuteness, and opportunities are not seldom taken to digress from strict description to collateral—perhaps too vivid delineation. If the author had headed his book, "Physiology of Marriage," and addressed it to the profession—or, at all events not specifically addressed it to the Public, we should not have adduced the slightest stricture on the work; but when we find the following passage in the Preface, we consider ourselves bound to animadvert on the tendency of the publication.

"A philosophical, social, *physical*, and *medical* history of the reproductive function of the vegetable and animal kingdoms, and of the abuses and disorders resulting from it in the latter, will, it is hoped, prove instructive and interesting to the *majority of general*, as well as medical readers."

That it will prove *interesting* to general readers, we have no doubt—and that it will convey *knowledge* to most of them, which they would be just as well without, we are quite satisfied. The work, in fact, is perfectly well adapted for medical perusal—except in the over-minuteness of details respecting Generation. If these details are unnecessarily minute for the professional reader, how unfit must they be for the eye and the imagination of the community at large—and especially of the female sex!!

As the work will have a prodigious sale, we sincerely advise the author to strike out all the minute details of his favourite subject, "the generation of animals," and fill up the space with moral and physical disquisitions that shall not shock the eyes and ears of the general reader.

---

Observations on Sciatica, and other Neuralgic Affections, &c. &c. together with an Account of the Waters of Bagneres and Barege, &c. By *Richard Carmichael*, M.R.I.A. 8vo. pp. 30. Dublin. April, 1838.

We are very sorry to find that our talented friend and countryman *has been* a victim to neuralgia—and to what is, perhaps, worse—dyspepsia. But half of our regret is dissipated by the good which has flowed from our author's sufferings. Many of the most valuable contributions to medical science owe their origin to the afflictions of individuals in the profession. Sydenham, Bree, and twenty other persons might be pointed out, in illustration—and now Mr. Carmichael is added to the list. There is, in this paper, a good deal of bold speculation, some original thinking, and a great many practical hints and observations —all conveyed in a discursive, and broken narrative, that is often entertaining, and sometimes even humorous. Indeed our candid author freely admits that his paper "is a melange which treats *de omnibus rebus, et quibusdam aliis.*" Notwithstanding this admission, we hope there are a few "*quibusdam aliis*" yet in reserve from the same pen.

Mr. C. has a hobby, as will appear in the sequel. He will never die of hydrophobia, if he were bitten by twenty mad dogs. But we would not swear that he will never need a strait-waistcoat, on account of a complaint which we may christen hydromania. He is a veritable disciple, too, of Abernethy—attributing most of our ills to the *chylopoietic organs*. He traces the origin of his own disorders to "inattention of taking nourishment at regular and fixed periods." He used to fast from eight in the morning till eight in the evening—certainly too long a period. In October 1827, he was seized with acute pain in the region of the stomach, attended with a sense of weight and distention, extending towards the spine. The extremities became cold, the face pale. This spasm lasted some hours, but gave way to hot brandy and water, with opium. Similar attacks followed; but in November, he experienced a dangerous gastric fever, with incessant sickness, gastralgia, delirium, &c. This fever was followed by great languor, and depression of spirits, though his professional prospects were most flourishing. He was apprehensive of diabetes, his urine being sometimes sixteen pints daily. "On examination, however, the urine was found to be *only* albuminous." We think that such a condition of urine was quite enough to cause some alarm, especially when attended by a general bad state of health. The warm-bath, however, brought the skin into action, and the diabetes disappeared. Here Mr. C. enjoins a strict attention to the state of the urine; for, whenever it varies from the normal condition, especially when it becomes "dark, clouded, and turbid," the patient has eaten or drunk more than was prudent, or used food whose quality disturbed the digestive process—or, that he has prolonged his fasting too far—or exercised his body or mind too much. This may be true, but it does not follow that the urine will always be sensibly changed by the above causes. We doubt the strict letter of the following passage :—

"In fact, the state of the urine points out when errors are committed by the dyspectic against the organic laws, with the same promptitude as the fabled ring of the Sultan Amurath indicated those against the moral laws." 6.

But to return. Although our author greatly improved in health, the slightest deviation from rigid temperance—the ingestion of vegetable food—or of more than a third in quantity of what used to be his allowance, brought on attacks of spasms and pain at the pit of the stomach, extending to the spine. A suspicion of gall-stones now arose in his mind, corroborated by a yellow appearance of the conjunctiva and even the skin. The attacks usually came on about two or three hours after food, and were only relieved by large and repeated doses of opium. This state went on for two years, and great debility and emaciation were the consequences. He now theorized a little, and came to the conclusion that the advent of chyme in the duodenum set the gall-bladder and ducts into activity in discharging the bile into the bowels—and that, as the secretion was viscid, the pain was occasioned by the expulsive efforts. From some little experience and observation, we venture to question this theory, and to suggest that these attacks were caused by the presence of food or chyme in the stomach or duodenum, whose mucous membrane was in a state of chronic inflammation, and whose nerves were in a condition of abnormal irritability and sensibility.

It was in the Summer of 1828 that sciatica was added to the long list of dyspeptic afflictions under which our author laboured. He hoped to subdue it by exercise—but this only increased its severity. He was obliged to be carried up stairs to see his patients, and, he candidly acknowledges that he madly persevered in the toils of his profession, long after his health had given way, and when he ought to have been seeking recovery in other climes—or, at all events, undisturbed by surgical practice. Fortunately Mr. C. is entirely independent of his profession, and is, therefore, the less excusable for sacrificing his health in the vortex of avocation.

Confined, at length, to his bed, Mr. C. tried a variety of remedies for the sciatica, such as mercury to salivation—turpentine to strangury—carbonate of iron—sarsaparilla—acupuncture, and all kinds of counter-irritation, but nothing gave relief, except large doses of opium, in the shape of black-drop. But the stomach and constitution became deranged in proportion to the ease procured by the opium. As a last resource, our author was advised to repair to the Pyrennees, and try the waters of Bagnères and Barèges. The voyage to Bourdeaux was prosperous and beneficial—and the only medicine which he took in that city was allopathic doses of La Fitte and Chateau Margeau—prescribed by an anti-Hahnemanic practitioner—Dr. Millengen. Arrived at Bagnères, our author commenced drinking the Lascere spring—a pint, at the temperature of 96 degs.—going into the warm bath, where he remained an hour daily—and imbibed the same quantity on coming out. These waters contain a variety of saline ingredients, but in such minute quantities as would rejoice the heart of Hahneman himself, when assured of the infinitessimal dose and gigantic efficacy of the remedy. In about 25 quarts of the water there are 71 grains of soda, magnesia, lime, &c. Notwithstanding this, our author, "derived decided, and almost immediate benefit from their use."

"The appetite improved, the tongue became clean; there was no longer a painful sense of distention, even after a slight repast. The bowels became regular; and the urine, which had been constantly turbid and loaded with a deep lateritious sediment, became clear, of a healthy amber colour, and considerably increased in quantity. The neuralgic pains now seldom required the tranquillizing influence of opium, and I had the pleasure of enjoying a sound sleep every night. We, therefore, must attribute the virtues of these waters not only to their temperature, but to their unknown state of combination. In no instance are we authorized to estimate the utility of mineral waters by the quantity or quality of their saline ingredients, of which the Lascere spring of Bagnères is a sufficient proof." 20.

The surprising efficacy of the Bagnères' waters induced our author, since that time, to try the powers of an artificial substitute, in dyspeptic cases, with turbid urine—namely,

"A small proportion of neutral salts, dissolved in a large quantity of warm-water, to be taken every morning, an hour or two before breakfast; during which time I enjoin some exercise either on foot or horseback. The ingredients of my saline powders are, from twenty to thirty grains of the bi-carbonate of soda, to sixty or ninety grains of Rochelle salts—the tartrate of soda and potash; to this, if a chalybeate is indicated, a couple of grains of the sulphate of iron is added. This I direct my patient to take in half-a-pint, or even a pint, of warm-water, (temp. 90 to 94), if his stomach can bear it, which it usually can, if divided into two doses, allowing an interval of half-an-hour between each. Now this simple medicine, with three or four grains of blue-pill every night, or every second night, according to the state of the biliary secretion, with due attention to diet, I have found of more advantage in dyspeptic cases, attended with turbid urine and lateritious sediments, than all the farrago of tonic and bitter medicines usually resorted to." 21

These have got the name of "Carmichael's Powders," but they are transmogrified into draughts, and tinged with tincture of cardamom.

Mr. C's improvement was so rapid that, in a fortnight he was able to ascend the mountains to *Barége*, situated at an elevation of more than four thousand feet above the level of the sea. There the waters are sulphureous, and at a temperature varying from 100 to 135 of Farenheit. They are too nauseous for internal use, but the *douche* is almost omnipotent. The visitors are numerous, and the accommodations wretched. Mr. C. was obliged to wait a fortnight before he could get a dip in Medea's Cauldron; then he was well nigh scalded to death.

"Eleven o'clock each night was the time appointed, and I considered myself fortunate to be permitted to use it even at this inconvenient time. At the appointed hour every night, I found a chaise-a-porteur at my lodging; in this I was conveyed in a few minutes to the douche. By the light of a glimmering lamp I found myself in a cell or dungeon, which appeared to be cut out of the rock; it was, however, so hot with sulphureous vapour, that at first I felt nearly suffocated, and I was glad to disencumber myself as quickly as possible of my clothes, in which I was assisted by a surly, grim, old attendant, who seemed naturally to appertain to a place filled with fire and brimstone. As soon as I was stretched upon a mattress which lay upon the floor, he turned a large cock about ten feet from the ground; the water, which was at the temperature of 120 deg. Farenheit, fell therefore with considerable force, and such was the shock which I at first felt, that I could scarcely refrain from crying out; however, I summoned resolution, and bore it for fifteen minutes, which my attendant said was the longest period that any person had been able to suffer under this infliction, ten minutes being the usual time. By shifting my position, I was enabled to let the water fall in succession over the entire of the trunk of the sciatic nerve and those branches which had been most painful. I found it very fatiguing as well as warm-work, and that I really required the assistance of my grim attendant to put on the flannel dresses with which I had come provided. This being done, I was immediately reconveyed by the same machine into my bed-room, where, without taking off my flannel dress, I got into bed between the blankets, and in a few minutes afterwards was covered with a profuse perspiration, which continued for four or five hours, and was promoted by drinking freely of some mild warm beverage. As soon as it had ceased, I changed my flannel dress for another, and remained in bed for several hours afterwards." 26.

In a fortnight after this, the descent of rain and snow warned Mr. C. that it was time to depart. He made some delightful excursions, on horseback, among the defiles of the Appennines, and returned a new man to the Emerald Isle, where we hope he may live "a thousand years," to use an Oriental expression, in health and happiness—for of wealth he is already in possession.

Making ample allowance for the elasticity of feeling, and the involuntary exaggeration which invariably mingle with our descriptions as to the salutary effects of mineral waters, when we recover health after a long illness, yet there is quite sufficient in the foregoing narrative to induce us to give a trial, in inveterate cases, to a remedy which is now easy of access, in consequence of the facilities afforded by steam navigation. We have an idea that change of scene and air, together with the moral and physical impressions which our author experienced, contributed not a little, in this case, to the felicitous results above enumerated—and that a trip up the Rhine, to Wisbaden, Baden Baden, and the German spas in general, would have been attended with effects, not very dissimilar to those that happily occurred in the vallies of the Pyrennees.

---

The Alternative: Disease and Premature Death; or, Health and Long Life. By *John Pinney*, Esq. Highley, London.

Philosophers have been much puzzled for a definition of man. We once thought we had made a notable discovery by characterizing him as a "suicidal animal." But, alas! the scorpion crossed our path, and claimed equality with the Lord of the Creation in this glorious prerogative! It is to be borne in mind, however, that the scorpion only destroys his life when he has no other way of escaping his enemy; whereas man commits suicide in the most leisurely and even scientific way imaginable—quite in the slow aqua tofana line—spreading the act over a space of many years. Thus he consumes bread with alum or bone-dust in it—wine with lead—ale with cocculus indicus—water taken from the common sewers—tea crisped and coloured with copper—air reeking from a thousand lungs—every eatable and drinkable, in short, more or less poisoned, and pernicious to health. But this is not the worst of it. The natural strength of his constitution might overpower these minute doses of poison; but he commits daily *intemperance* in all these necessaries or luxuries of life. He eats and drinks twice as much as he requires—burns arsenicated candles when he ought to be in bed—and lies in bed four or five hours after the sun has summoned him from his close and air-polluted chamber! Against these and a thousand other physical ills and evil habits he has been warned by divines, philosophers, and physicians, from Plato down to our present author Pinney—and all in vain! The latter writer, though a lawyer by profession, has composed a volume of excellent didactic rules and precepts for maintaining health, and attaining extreme old age. They have only one fault—that the world cannot or will not put them in practice! Mr. Pinney is not at all of Juvenal's opinion, who avers that—

"Few
Know their own good, or knowing it pursue."

He thinks that every man knows, or may know, everything that is necessary, or conducive to his health and happiness.

"Is it not within the power of man to observe and judge of the quantity and quality of his diet? To consume no more at his meals than Nature demands? To avoid inhaling a confined, impure, or otherwise unwholesome air? To take daily active exercise? To rise early in the morning, and to retire to rest early at night? To insure the proper discharge of sensible and insensible perspiration, by exercise and daily ablutions of the body? To refrain from taking deleterious medicine? and to pay strict regard to the alvine exonerations?"

Now to every one of these questions we might safely answer in the negative. The physician of 50 years' study and experience would not be able to judge accurately on all those points—and if so, how could the world, who have their various avocations and callings to pursue, and to occupy their time, be capable of determining such difficult problems in physiology, hygiene, and therapeutics? If every man might thus be his own doctor, we ask Mr. Pinney, why every man might not also be his own lawyer? Surely the mechanism and laws of the human economy are as difficult to unravel and ascertain, as those of the criminal code, or civil jurisprudence. But as the man who pleads his own cause has generally a fool for a client, so the patient who prescribes for himself, on all occasions, will seldom be out of the hands of an ignoramus as his doctor.

At page 18, Mr. Penny tells us that—"years *alone* do not destroy life."

"It is insidious disease, springing from the wearing influence of irritation on the body, by means of indolence and intemperance, that causes the thread of existence to be prematurely severed; and thus

"Man makes a death which Nature never made."

To this we demur. Indeed in the very next page, the author contradicts this position of immortality. "Notwithstanding the provisions made by Nature for correcting every derangement incident to the human frame, every one knows that it contains within itself the elements of its own dissolution."

One of the best sections in the book is that on "INDOLENCE" and "LUXURY"—a perusal of which may do some good among the lazy and voracious classes of society. The chapter on *exercise* is also praiseworthy. At page 56 he relates an anecdote of our late worthy friend, Dr. Uwins, which we could give a very different version to; but peace to the manes of our amiable, jocular, and most eccentric friend, and occasional antagonist in medical discussions!

The chapter on "MEDICAL REFORM" is the longest in the work; and although it contains some rather illiberal reflections on the profession, or rather some members thereof, yet there are some pertinent remarks in it, which are deserving of attention. We can only make room for one short extract.

"In my opinion, the only effectual remedy for this evil is, a direct interference of the Legislature, to protect the public not only against ill-educated and uninstructed practitioners, but also to correct the ineffectual modes adopted by the different governing bodies of the Royal Medical Colleges for ascertaining the proficiency of medical practitioners.

The constitution of the science and practice of medicine upon a secure and rational basis, might then be depended on; and this object might, I conceive, be most readily achieved, by investing persons of integrity and honour beyond the possibility of suspicion, with full authority to control and regulate medical education, *and fix one standard of qualification for the entire body of the profession.* I venture also to express it as my opinion, that the practice of medicine ought to be entirely separated from the business of compounding and dispensing drugs, which should only be furnished in the way of medicine by one not interested in the sale of them in that form."

We think that most of our readers will acknowledge that there is much truth in the foregoing passage. Even the proposal of having only one qualification for all the present classes or denominations of medical society is, like that of the Ballot, daily becoming more popular—and we have little doubt that, in less than twenty years, it will be the law of the land. Mr. Pinney makes some most *unjust*, and, except in a few very rare instances, *unfounded* remarks on the supposed confederacy between the physicians and surgeons and the general practitioners and chemists, to fill the pockets of all, and drench the stomachs of the public. Unhappily, the present disreputable mode of remuneration gives rise to, and supports this suspicion in the minds of the community. Mr. Pinney, like most other speculative people, proposes a remedy which, however, can never become general—an *annual* allowance to the general practitioner from individuals or families. The plan is in operation among the Israelites, of this metropolis; but, from what we have observed, it does not very well succeed—at least we have not seldom seen a NON-CONTRACTING doctor called in, when the disease assumed a serious character, indicating a want of confidence in him who was paid by the year instead of the visits.

---

TREATISE ON HERNIA: COMPRISING THE SURGICAL ANATOMY, OPERATIVE SURGERY AND TREATMENT OF THAT IMPORTANT DISEASE. By *Malcolm W. Hilles.* London, Henry Renshaw.

No disease, perhaps, requires a more thorough acquaintance with its pathology than hernia, not only from the frequency of its occurrence, but also from the great liability there exists of its subjecting the patient to a dangerous and troublesome operation. To the operating surgeon, a strict knowledge of the anatomy of the parts connected with this disease, is, from the little delay of which it admits, absolutely indispensable. The little work now under review does not pretend to be an elaborate treatise on hernia, but seeks merely to point out, in as concise a manner as possible, the surgical anatomy and treatment of that formidable disease.

Mr. Hilles sets out with the different divisions which have been made of this disease; he next proceeds to the consideration of inguinal hernia; under this heading our author first details the surgical anatomy of the parts, and then explains the operation for the relief of strangulated inguinal hernia; he also notices cursorily the various modifications which exist of this disease; this section occupies by far the largest portion of the book. The next chapter is devoted to femoral hernia, the anatomy of which Mr. Hilles describes fully and clearly. The remaining pages of the work are devoted to hernia in general, and to a detail of its causes and general treatment. This concludes the work, but in taking leave, it is but justice to the author to state that the book is carefully written, and contains much useful information, clearly and intelligibly conveyed.

---

PHYSICAL EDUCATION; OR THE NURTURE AND MANAGEMENT OF CHILDREN, FOUNDED ON THE STUDY OF THEIR NATURE AND CONSTITUTION. By *Samuel Smiles,* Surgeon. Edinburgh, Oliver and Boyd; London, Simpkin, Marshall and Co.

THE object of this work, according to the preface, is, to assist parents, "by familiarly describing the construction of the infant's frame, the functions of life, and the laws that regulate them, as well as the gradual development of the sound mind in the sound body, by means of a proper education and management; and pointing out a few of the more general causes of derangement, such as a mother's or nurse's care may easily avoid." The first chapter furnishes an account of the peculiarities of the infant's constitution; and order of its development. The consideration of the digestion of infants occupies the next section, which contains several useful hints to parents respecting the nutrition of children. The three succeeding chapters are devoted to the functions of respiration, depuration by the skin, and voluntary motion; the seventh and last contains a *resumé* of all the contents of the preceding pages.

It can scarcely be expected, from the small size of the work, that any thing beyond a mere outline of the subjects on which it treats, can be afforded; the account therefore of the infant constitution and of the physiology of the vital functions, is necessarily imperfect; this however is more the fault of the class to which the book belongs, than of the book itself. The work is written throughout in an easy familiar style, and is well worthy the attention of the general reader, for whom it is more immediately designed.

---

A PRACTICAL TREATISE ON FRACTURES. Illustrated with Sixty Wood-cuts. By *Edward F. Lonsdale,* Surgeon; Demonstrator of Anatomy at the Middlesex Hospital School of Medicine, and formerly House-Surgeon to the Hospital. 8vo. pp. 536, London, 1838.

MR. LONSDALE is a young gentleman of much professional zeal and ability. He has been at considerable pains to collect in one volume all that it is desirable to know with regard to the management of fractures, and his Treatise is one which is likely to be very useful. He has adopted the plan of illustrating the text by numerous wood-cuts, a plan which is daily growing more popular, and which is in itself very excellent.

It cannot be expected of us to offer any copious account, of a work so elementary as this must necessarily be. The order in which the subject is treated may be gleaned from the headings of the various sections. They stand thus. General Observations on Fractures—Of Particular Kinds of Fracture—Of Fractures of the Fore Arm—Of Fractures of the Humerus—Of Fractures of the Scapula—Of Fractures of the Clavicle—Of Fractures of the Bones of the Face—Of Fractures of the Bones of the Trunk—Of Fractures of the Femur—Of Fractures of the Patella—Of Fractures of the Leg—Of Fractures of the Bones of the Foot.

As a specimen of the manner in which our author writes, we may take his view of the cause of non-union of fractured neck of the thigh-bone internal to the capsule.

"In order to produce bony union in fractures external to joints, it is found by experiments that have been made of late years, and from observations on the human subject, that it is necessary for the soft parts round the ends of the bone to take a most active part in the process; in fact they perform the sole part during the first stage of it, while the fractured surfaces are merely lying in apposition, without any actual union between them. This action and change that takes place in the surrounding parts, has been described, when speaking of the union of fractures, as the provisional callus ; being essential for the formation of the capsule, which steadies the ends of the bone before they are actually united. Now we have only to consider the circumstances under which the neck of the thigh bone is placed when fractured, and when the capsular ligament is not torn, and it will be seen at once how different they are ; for now the fractured ends are completely isolated, and any change of action that may take place in the soft parts externally, can have no effect upon the ends of the bone themselves ; besides there is no bruising now of the cellular and muscular substance, as there is in fractures of the shaft of the bone, so that there is no disposition for the provisional callus to form, even were it possible for it to get at the ends of the bone. If then this first and most important part of the process cannot be performed, it is quite impossible that the ends of the bone themselves should unite, as the latter part is altogether dependant on the former. This, in my opinion, explains at once in a clear and simple way (without the necessity of having recourse to the want of apposition or rest, or to the little vascularity in the upper portion of bone), why it is that union by bone never takes place when the fracture is quite within the capsule, and the capsule itself not torn. I say never takes place, because I believe that the cases related in opposition to this assertion are not sufficiently unobjectionable to form a decided opinion upon; for, in the words of Sir Astley Cooper, already quoted, 'I cannot give credence to such cases, until I see that the authors were aware of the distinctions between fractures within and fractures external to the articulation.'" 382.

There cannot be a doubt of the partial correctness of Mr. Lonsdale's, or rather of John Bell's view, for he candidly quotes that surgeon's sentiments to the same effect. But neither can there be a question, that the view is only partial, and that the imperfect vascular supply of the bone, and the difficulty of procuring adequate rest, are also powerful co-operating causes. In this, as in many other controversies in physic, men have been so anxious to discover some one cause when there are several, that they have allowed some very palpable fact to pass unheeded.

There are many acute observations, and many valuable suggestions in this volume. It would be, however, an injustice to the author to pick out unconnected remarks, and we therefore recommend our young surgical readers to purchase the work itself. Mr. Lonsdale deserves every encouragement.

---

Experiments and Observations on the Gastric Juice, and the Physiology of Digestion, &c. By *William Beaumont*, M. D. Reprinted with Notes, by *Andrew Combe*, M.D. Edinburgh, 1838.

Dr. Beaumont's experiments have excited great interest in every country of the civilized world, and Dr. Combe has conferred a benefit on the profession of this country by the present reprint, and by many valuable notes appended to it. It is a work which will not admit of analysis ; but its size and price will render it easy of access to all who take interest in the subject, or indeed in their profession. But as Dr. Beaumont has drawn up a summary of the inferences to be drawn from his numerous experiments, these deserve as extensive a diffusion as a periodical can give them. These therefore we subjoin.

"1. That *hunger* is the effect of *distention* of the vessels that secrete the gastric juice.

2. That the processes of *mastication, insalivation,* and *deglutition,* in an abstract point of view, do not, in any way, affect the digestion of food ; or, in other words when food is introduced directly into the stomach, in a finely divided state, without these previous steps, it is as readily and as perfectly digested as when they have been taken.

3. That *saliva* does not possess the properties of an alimentary solvent.
4. That the *first* stage of digestion is effected in the stomach.
5. That the *inner coat* of the stomach, is of a pale *pink* colour, varying in its hues, according to its full or empty state.
6. That in health, it is constantly sheathed with a *mucous* coat.
7. That the natural *temperature* of the stomach is 100° Fahrenheit.
8. That the temperature is *not elevated* by the ingestion of food.
9. That *exercise elevates* the temperature; and that *sleep* or *rest*, in a recumbent position, *depresses* it.
10. That stimulating *condiments* are injurious to the healthy stomach.
11. That the use of *ardent spirits always* produces disease of the stomach, if persevered in.
12. That the appearance of the interior of the stomach, *in disease*, is essentially different from that of its *healthy* state.
13. That the *agent* of chymification is the *gastric juice.*
14. That the pure gastric juice is fluid, *clear*, and *transparent*, without *odour*, a little salt, and perceptibly *acid.*
15. That it contains free *muriatic acid* and some other active *chemical* principles.
16. That it is never found *free* in the gastric cavity; but is always excited to discharge itself by the introduction of *food*, or other irritants.
17. That it is secreted from vessels distinct from the mucous follicles.
18. That it is seldom obtained pure, but is generally mixed with mucus, and sometimes with saliva. When pure it is capable of being kept for months, and perhaps for years.*
19. That it *coagulates* albumen, and afterwards *dissolves* the *coagulæ.*
20. That it *checks* the progress of putrefaction.
21. That it acts as a *solvent* of food, and alters its properties.
22. That like other chemical agents, it *commences* its action on food, as soon as it comes in *contact* with it.
23. That it is capable of *combining* with a certain and fixed *quantity* of food, and when more aliment is presented for its action than it will dissolve, disturbance of the stomach, or 'indigestion,' will ensue.
24. That its action is facilitated by the *warmth* and *motions* of the stomach.
25. That it is *invariably* the *same substance*, modified only by *admixture* with other fluids.
26. That it becomes intimately *mixed* and *blended* with the ingestæ in the stomach, by the motions of that organ.
27. That *no other* fluid produces the same effect on food that gastric juice does; and that it is the *only solvent* of *aliment.*
28. That *gentle exercise* facilitates the digestion of food.
29. That *bile* is not ordinarily found *in the stomach*, and is *not* commonly *necessary* for the digestion of food; but
30. That, when *oily* food has been used, bile assists its digestion.
31. That the action of the stomach and its fluids are the same on *all kinds* of diet.
32. That the *time* required for the digestion of food is various, depending upon the quantity and quality of the food, state of the stomach, &c.; but that the time ordinarily required for the disposal of a moderate meal of the fibrous parts of meat with bread, &c., is from three to three and a half hours.

---

* "I have now (Nov. 1, 1833) in my possession, some clear gastric juice, possessing all its original properties, unchanged and undiminished, which was taken from the stomach in Dec. 1832, about eleven months ago, and has been kept tightly corked in vials."

33. That the *digestibility* of aliment does not depend upon the *quantity* of nutrient principles that it contains.

34. That the susceptibility of digestion does not, however, depend altogether upon *natural* or *chemical* distinctions.

35. That *bulk*, as well as *nutriment*, is necessary to the articles of diet.

36. That digestion is facilitated by *minuteness* of *division* and *tenderness* of *fibre*, and retarded by opposite qualities.

37. That *solid* food, of a certain texture, is easier of digestion than *fluid*.

38. That *animal* and *farinaceous* aliments are more easy of digestion than *vegetable*.

39. That *oily* food is difficult of digestion, though it contains a large proportion of the nutrient principles.

40. That the *quantity* of food generally taken, is more than the wants of the system require; and that such excess, if persevered in, generally produces not only functional aberration, but disease of the coats of the stomach.

41. That the *ultimate principles* of aliment are always the same, from whatever food they may be obtained.

42. That *chyme* is *homogeneous*, but variable in its *colour* and *consistence*.

43. That towards the *latter stages* of chymification, it becomes more *acid* and *stimulating*, and passes more rapidly from the stomach.

44. That *water*, *ardent spirits*, and most other *fluids*, are not affected by the gastric juice, but pass from the stomach soon after they have been received.

45. That the motions of the stomach produce a constant *churning* of its contents, and *admixture* of food and gastric juice.

46. That these motions are in two directions, *transversely* and *longitudinally*.

47. That the *expulsion* of the chyme is assisted by a *transverse band*, &c.

48. That *chyle* is formed in the duodenum and small intestines, by the action of *bile* and *pancreatic juice* on the chyme.

49. That crude *chyle* is a *semi-transparent whey-coloured* fluid.

50. That it is further changed by the action of the *lacteals* and *mesenteric glands*. This is only an *inference* from the other facts. It has been the subject of experiment." 302.

It will be obvious that a vast majority of these inferences were come to by observation and experience, long before accident disclosed to Dr. Beaumont the secret operations of nature in the process of digestion. Notwithstanding the facilities which Dr. B. enjoyed, we have some doubts as to the truth of his inferences. Thus the very first is very questionable; for it is far more likely that the state of the nerves rather than turgidity of the vessels produces the sense of hunger. In respect to the effect of gentle exercise after food as accelerating digestion, the fact may be true in a healthy stomach; but we are quite certain that, in dyspepsia, rest, or at the most passive exercise in an easy carriage, is infinitely more propitious to digestion than pedestrian or other active exercise. We quite agree with the author in his 40th inference, and have often pointed it out before Dr. Beaumont wrote.

### NATURAL HISTORY.

I. A History of British Birds. By *William Yarrell*, F.L.S. Secretary to the Zoological Society. Illustrated by a Woodcut of each species, and numerous Vignettes. Parts IV. and V. Price 2s. 6d. each Part.

II. A History of British Reptiles. By *Thomas Bell*, F.R.S. F.L.S. Professor of Zoology in King's College, London. Illustrated by a Woodcut of each Species, with some of the Varieties, and numerous Vignettes. Part I. Price 2s. 6d.

THE delightful study of Natural History is daily growing on the tastes of the intelligent portion of the public. The zoological collections that are formed, or in process of formation, in the larger towns of the empire, both spring from this taste and will extend it. The same may be said of works like those before us—works produced under the superintendence of gentlemen of high attainments—and calculated to diffuse an acquaintance with the animals of our own country.

We have spoken on several occasions of a former work of the same description—the History of British Quadrupeds. That has been for some time completed, and may be found, we trust, in the library of many of our readers, as we have taken care to place it in our own. The works before us may be said to be sequels to that. They serve to complete the history of British animals.

Parts IV, V, and VI, of the History of British Birds, by Mr. Yarrell, are quite equal to their predecessors. The descriptions are accurate and lively—the woodcuts of the species graphic,—and the vignettes pretty. We cordially recommend a work so well commenced, and so certain to be carried on. We shall take care to notice the successive Parts as they appear, and to keep them before the eyes of our readers.

We have as yet received only Part I of the History of British Reptiles. We presume that Part II. has not yet been published. The following extract from the Prospectus will explain the views of Mr. Bell in commencing it.

"In pursuance of the plan already commenced by the publication of the History of British Fishes and of British Quadrupeds, it is intended that the latter work, now completed, shall be immediately succeeded by a History of British Reptiles, by the same author; which, with the former works, and that of Mr. Yarrell on British Birds, will complete the Natural History of the Vertebrate Animals of the British Islands.

The Reptiles of this country, although few in number, are not devoid of considerable interest: their habits are popularly much misunderstood, and several innocent and useful species are shunned and destroyed from a mistaken notion that they are directly or indirectly noxious to man. The elucidation of their habits, the distinctive description of the species, their geographical distribution, and the history of the transformation of all the amphibious forms, are amongst the subjects which will be fully discussed.

The author has already to offer his thanks for the promise of much valuable assistance, particularly from a well-known zealous naturalist of Ireland, whose communications on the Reptiles of that portion of the kingdom cannot but be highly interesting; and he solicits from all who are engaged in the pursuit of British Zoology the continuance of the kind and liberal attention which he has already so extensively received during the progress of his former work.

The woodcuts will be numerous, corresponding in their general style with those of the preceding works; and in addition to a figure of each species, and of some of the most important varieties, will comprise many illustrations of structure and development, particularly the transformation of the Batrachian genera."

We need not add our conviction that the work, indeed the whole series of works, deserve the encouragement of the public, and especially of the profession. Mr. Bell's name is a guarantee for the accuracy and the ability of the execution. The Part before us is very good.

### WORKS ON ANATOMY.

I. A Series of Anatomical Plates, &c. By *Jones Quain*. Division III. Nerves. Fasciculi L. to LX.

We have so often had occasion to notice these Anatomical Plates, and with such commendation, that it is difficult to vary the language of approbation. The fasciculi before us quite maintain the reputation acquired by their predecessors; and the plates of the nerves bid fair, for they are not yet complete, to prove very valuable to the anatomical student. Taking into consideration their size, execution, and price, there are few students who need or who ought to be without either a part or the whole of the work. When the division of the nerves is completed, and we trust that it will be a very copious one, we shall take care to apprize our readers of it, and to revert to the Plates. We wish them all success, and recommend them strongly.

II. Velpeau's Anatomy of Regions. Translated from the French. By *Henry Hancock*, Lecturer on Practical and Surgical Anatomy at the Westminster Hospital School of Medicine, &c. 8vo. pp. 565. London, 1838.

Mr. Hancock informs us, in a brief advertisement, that,

"In preparing the following treatise, it has been my desire to furnish the student with a complete work on the Anatomy of Regions, restrained within such limits as would make it available to all members of the profession. This will account for the omission of the plates, and that portion of the original which treats of General Anatomy. It has, also, been my endeavour, whilst I rendered the translation as literal as the various idioms permitted, to give the descriptions as clearly and simply as possible."

It is not necessary for us to speak of the merits of the original. M. Velpeau's Anatomy of Regions decidedly occupies the first place among works on that important subject. The anatomy of regions is little attended to in this country. Allan Burns has written a very excellent treatise on the surgical anatomy of the neck, but that may be said to be the only monograph on regional anatomy in our language. Mr. Harrison's surgical anatomy of the arteries is excellent, but that only treats of bits of regions, and of the immediate neighbourhood of the great arterial trunks.

We have in our possession an American Translation of M. Velpeau's work. But that is scarce in England, and is more voluminous than Mr. Hancock's. The latter Gentleman has therefore rendered a service to our medical students and surgeons, by presenting to them, in a convenient form, a complete treatise on regional anatomy. He has executed his task with fidelity and judgment, and the work should be in the possession of every anatomical student. We strongly recommend it.

III. The Anatomy of the Regions, interested in the Surgical Operations performed upon the Human Body, with occasional Views of the Pathological Conditions which render the Interference of the Surgeon necessary. In a series of Plates engraved, on India paper, the size of life. By *J. Lebaudy*, M.D. With additions, price £1 4s. Bailliere, 1838.

We take shame to ourselves for not having noticed this meritorious work before. It escaped us. M. Bailliere has displayed his usual spirit, in publishing, at a reasonable rate, the work of M. Lebaudy in an English form. Indeed we may take this opportunity of directing the attention of our readers to M. Bailliere himself. His civility, attention to orders, and liberality, ought to be generally known in the profession. He deserves every possible encouragement, both private and public. But this by the way.

The work before us contains eighteen admirably executed Plates, devoted to the exhibition of—1, the Hyo-Cardiac Region—2, the Occipito-Clavicular Region—3, the Sub-maxillary Region—4, the Supra-Clavicular and Cervical Region—5, the Left Superior Thoracic and Inferior Cervical Regions—6, the Internal Brachial Region—7, the Bend of the Arm—8, the same deep—9, the Anti-Brachial Region—10, the Hand of a Subject who died of Tubercular Phthisis—11, the Fœtus in the Sixth Month—12, the Superficial Inguinal Region—13, the Deep Inguinal Region—14, Femoral and Inguinal Hernia—15, Femoral Hernia, the Sac laid open—16, Inguinal Hernia on the left side—17, the Superficial Popliteal Region—18, the Consequences of Burns.

It must be evident that these plates represent an important and considerable portion of the anatomy of regions. They are executed not only with clearness, but with accuracy, and are calculated to be highly useful. The letter-press accompanying them is not only sufficiently explanatory of them, but likewise points out the best methods of performing those operations which most properly belong to the several regions.

IV. A Series of Anatomical Sketches and Diagrams. With Descriptions and References. By *Thomas Wormald*, Assistant-Surgeon and Demonstrator of Anatomy at St. Bartholomew's Hospital, and *Andrew Melville Mc Whinnie*, Teacher of Practical Anatomy at St. Bartholomew's Hospital, and late House Surgeon to that Institution. Highley, London, 1838. Price 4s.

This Part contains five Plates. They are light and easy sketches, intended as an introduction to the more elaborate systems of plates. In point of fact, the gentlemen before us have published some of the diagrams which most modern teachers are in the habit of employing for the instruction of their pupils. They are calculated to serve the purpose for which they were designed.

